# Validation of an automated total and viable cell counting for GMP-compliant production of human bone marrow stromal cells

**DOI:** 10.1016/j.btre.2026.e00974

**Published:** 2026-07-27

**Authors:** Scarlett Desclaux, Narong Chitchongyingcharoen, Chanatat Threeratanah, Chavalwit Soonthronsuk, Sorawut Thamyongkit, Chagriya Kitiyakara, Natnaree Siriwon

**Affiliations:** a Chakri Naruebodindra Medical Institute, Faculty of Medicine Ramathibodi Hospital, Mahidol University, Samut Prakan, Thailand; b Advanced Cell Therapy and Biomedical Device Manufacturing Center, Faculty of Medicine Ramathibodi Hospital, Mahidol University, Samut Prakan, Thailand; c Division of Nephrology, Department of Medicine, Faculty of Medicine Ramathibodi Hospital, Mahidol University, Bangkok, Thailand

**Keywords:** Total cell concentration, viability, Automated cell counter, BMSCs, GMP manufacturing

## Abstract

Bone marrow stromal cells (BMSCs) are classified as Advanced Therapy Medicinal Products (ATMPs) in the subcategory of Cell Therapy Medicinal Products (CTMPs), and the production of these cells for clinical use under good manufacturing practice (GMP) standards is regulated by the European Medicines Agency (EMA). To meet GMP standards for CTMPs, the accuracy and precision of cell-counting methods are essential for monitoring in-process quality and controlling clinical dosing. To achieve this purpose, we validated an automated cell-counting method for evaluating cell concentration and viability, enabling large-scale analysis and reducing analyst-associated variability. Automated cell counting was performed using the LUNA™ automated cell counter, and we validated the parameters against the hemocytometer using the ICH Q2R1 framework. The results showed that automated cell counting yielded acceptable accuracy (90–110%) and RSD (<10%). This demonstrates that LUNA™ automated cell counters may provide an efficient alternative for measuring BMSC cell concentration and viability.

## Highlights

• We have established a BMSC counting protocol with defined parameters for evaluating total cell concentration with the LUNA^TM^ automated cell counter.
• The LUNA^TM^ automated cell counter showed accuracy, precision and linearity comparable to the manual cell-count method.

## Graphical abstract

**Figure:**
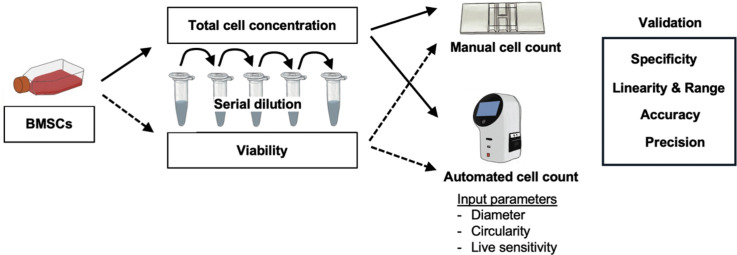

## 1 Introduction

Adult mesenchymal stem cells (MSCs) have emerged as a potential cell source for regenerative medicine due to their multilineage differentiation ability and high proliferative potential. MSCs have been successfully applied for a wide range of disorders due to their unique regenerative, angiogenic, and anti-inflammatory properties [[1], [2], [3], [4]]. Currently, MSCs are classified as advanced therapy medicinal products (ATMPs) under European Union Regulation (EC) 1394/2007 and must be manufactured in compliance with Good Manufacturing Practices (GMP) to ensure the consistency of product quality and efficacy [5,6]. Cell counting is a measurement of cell quantity and has been widely used to monitor cell proliferation rates and seeding cells during the MSC manufacturing process, as well as to determine the cell dosage of the final product before batch release [7]. Therefore, selecting an accurate cell-counting method is essential.

The conventional method is direct cell counting by the operator using a hemocytometer. Although manual cell counting is an accepted reference method described in the European Pharmacopeia (EP), this method is time-consuming and often subject to operator-dependent variability. To reduce heterogeneity in results and analytical time, automated cell counting offers a more advantageous approach [8]. The GMP-compliant automated cell counting systems are widely used in ATMP manufacturing to substitute the manual cell counting method [[8], [9], [10]]. Particularly, NucleoCounter® and Vi-CELL™ XR have been demonstrated to be suitable platforms for the manufacturing of the MSC-based ATMPs [10,11]. One of the commercially available automated cell counting systems, LUNA-II™ automated cell counter from Logos Biosystems is used in applications ranging from basic cell maintenance to rigorous control the quality of procedures in diverse cell types with cell concentration range between 5 × 10^4^ and 1 × 10^7^ cells/mL in which the parameter setting is customized accounting for minimum and maximum cell diameter, circularity, and sensitivity. For batch release purposes, the LUNA-II ™ automated cell counter has key GMP compliance features, which include 21 CFR Part 11 compliance, validation protocols for installation and operational qualification, and contamination control through the use of disposable slides. It is a more economical option than other machines, such as the NucleoCounter®, while also complying with GMP requirements.

Total cell concentration and viability are two critical quality attributes (CQAs) that serve as controls for in-process quality monitoring and product batch release. The LUNA-II™ Automated Cell Counter is an image-based cell counting system that combines brightfield microscopy with automated image analysis. For viability assessment, cells are mixed with trypan blue dye before being loaded into the counting chamber. Viable cells exclude trypan blue and remain unstained, whereas non-viable cells take up the dye and appear blue. The instrument automatically captures cell images and applies image-processing algorithms to identify, classify, and count live and dead cells, thereby determining total cell concentration and viability [12]. Alternatively, NucleoCounter® and Cellometer™ automated cell counters have emerged as automated fluorescent-based cell counting using nucleic acid-binding dyes such as acridine orange/propidium iodide (AO/PI) in addition to flow cytometry with 7-amino actinomycin D (7-AAD) or PI staining [8,13]. The suitability of each cell counting method has to be determined through validation studies according to the International Conference on Harmonization (ICH) Q2R1 Guidelines and EP (10th ed. nucleated cell count and viability (2.7.29)) [14,15]. The validation characteristics proposed in ICH Q2R1 focus on specificity, linearity, range, accuracy, and precision [14,16]. According to the ICH Q2R1 framework, (i) specificity is evaluated on the media or buffer used for cell preparation that may interfere with the image analysis in the automated method; (ii) accuracy is the proximity of the measurement results to the true cell concentration (obtained from a reference method); (iii) precision is the closeness between the results from multiple runs of the same homogeneous sample; (iv) linearity is its ability to obtain results that are directly proportional to the concentration of analyte in a linear fashion; and (v) range is the interval value between the upper and lower concentration of analyte [14]. Therefore, the aim of the present study is to validate the performance of the LUNA™ automated cell counting method relative to the manual hemocytometer, focusing specifically on the determination of cell concentration and viability during GMP-compliant MSC production.

## 2 Materials and methods

### 2.1 Cell culture and cell count reagents

Bone marrow stromal cells (BMSCs) were obtained from bone marrow aspirates of patients who underwent hip fracture surgery at Ramathibodi Chakrinaruebodindra Hospital. This study has received ethics approval from the IRB of the Faculty of Medicine Ramathibodi Hospital, Mahidol University (MURA2025/186), approved on March 3, 2025. Informed consent to collect bone marrow aspirates were obtained from patients prior to the procedure. All research activities complied with ethical regulations and were conducted in accordance with the hospital's regulations. PBS (Dulbecco’s phosphate-buffered saline, 1X pH 7.4) was acquired from Gibco1-Life technologies™ (Waltham, MA, USA). MSC NutriStem® XF medium, supplement mix and recombinant, trypsin EDTA solution were purchased from Sartorius (Goettingen, Germany). 10% PLTGold® was obtained from Mill Creek Life Sciences (Rochester, MN, USA). Soybean trypsin inhibitor and Lymphoprep™ were purchased from STEMCELL™ Technologies (Vancouver, BC, Canada). 0.4% trypan blue solution was acquired from Sigma–Aldrich (St. Louis, MO, USA). Hanks’ balanced salt solution (HBSS) was obtained from Thermo Fisher Scientific (Waltham, MA, USA). 5% human serum albumin (HSA) was acquired from Grifols (Frankfurt, Germany).

### 2.2 Preparation of cell solutions

Bone marrow aspirates were collected in heparin-coated tubes. Afterwards, bone marrow mononuclear cells were isolated through centrifugation using a Lymphoprep™ density gradient and then selected through the process of plastic adherence. Cells were then cultured at density of 2–5 × 10^3^ cells/cm^2^ in NutriStem® MSC XF medium containing supplement mix and 10% PLTGold® in CellBind® surface 75 cm^2^ flask and Hyperflask (Corning Incorporated, New York, NY, USA) at passage 1 and 2, respectively and incubated at 37 °C in 5% CO_2_. The medium was subsequently changed every 3 to 4 days. Once 80–90% confluency was reached, BMSCs were washed twice in PBS, trypsinized, and incubated for 5 min. The BMSCs were then neutralized with soybean trypsin inhibitor and centrifuged at 800 x g for 5 min. The BMSC pellets were washed twice with HBSS in a final volume of 40 mL before being re-suspended in 2.5 mL of 5% HSA and filled into syringe as a fresh cell product. The target dose is 8 × 10^6^ cells/mL. Freshly prepared BMSCs were then re-suspended in appropriate volume of HBSS to prepare a stock cell solution with an estimated total cell concentration. The total cell concentration was estimated by the manual cell count using a hemocytometer. For cell viability, the serial dilutions of a mixture containing the non-viable cells and viable cells from the stock cell solution were created. The non-viable cells were prepared by incubating at 90 °C for 10 min, and confirmed cell death by the presence of fully blue-stained cells in the hemocytometer.

### 2.3 Manual cell counting

Manual cell count was performed using an Improved neubauer cell counting chamber consisting of nine squared wells, each with a 1 mm^2^ area. The BMSC suspensions were diluted 1:1 with 0.4% trypan blue in new centrifuge tubes. 10 μL of mix was loaded into each counting chamber of the hemocytometer for a total of two samplings. The BMSCs in the large squares at the four corners were counted and excluded when they touched either the bottom or right line. Cell counts were performed under a bright-field microscope with a 10X objective (Nikon Eclipse Ts2; Nikon, Tokyo, Japan). The total cell numbers, including viable and non-viable cells, were counted. Remarkably, an average of <10 cells per corner grid of the hemocytometer (approximately the concentration of 2 × 10^5^ cells/mL) would be too low to count accurately.

### 2.4 Automated cell counting

A mixture of the BMSC suspension and 0.4% trypan blue was prepared prior to loading 10 μL into each chamber of the cell counting slide, for a total of 2 samplings. The parameter settings for BMSC counting included minimum diameter (3–10 µm), maximum diameter (30 µm), circularity (50, 75, 100%), and live sensitivity (1–9). Cell diameter parameters refer to the size of cells that include in the count. Circularity parameters measure the roundness of cells and include/exclude cells based on shape. Live sensitivity parameters adjust how the machine detects live cells based on the brightness of the cell center. The total cell, viable, and non-viable cell numbers were assessed using a LUNA-II™ automated cell counter (Logos Biosystems, Anyang, South Korea).

### 2.5 Validation designs

**Fig. 1:**
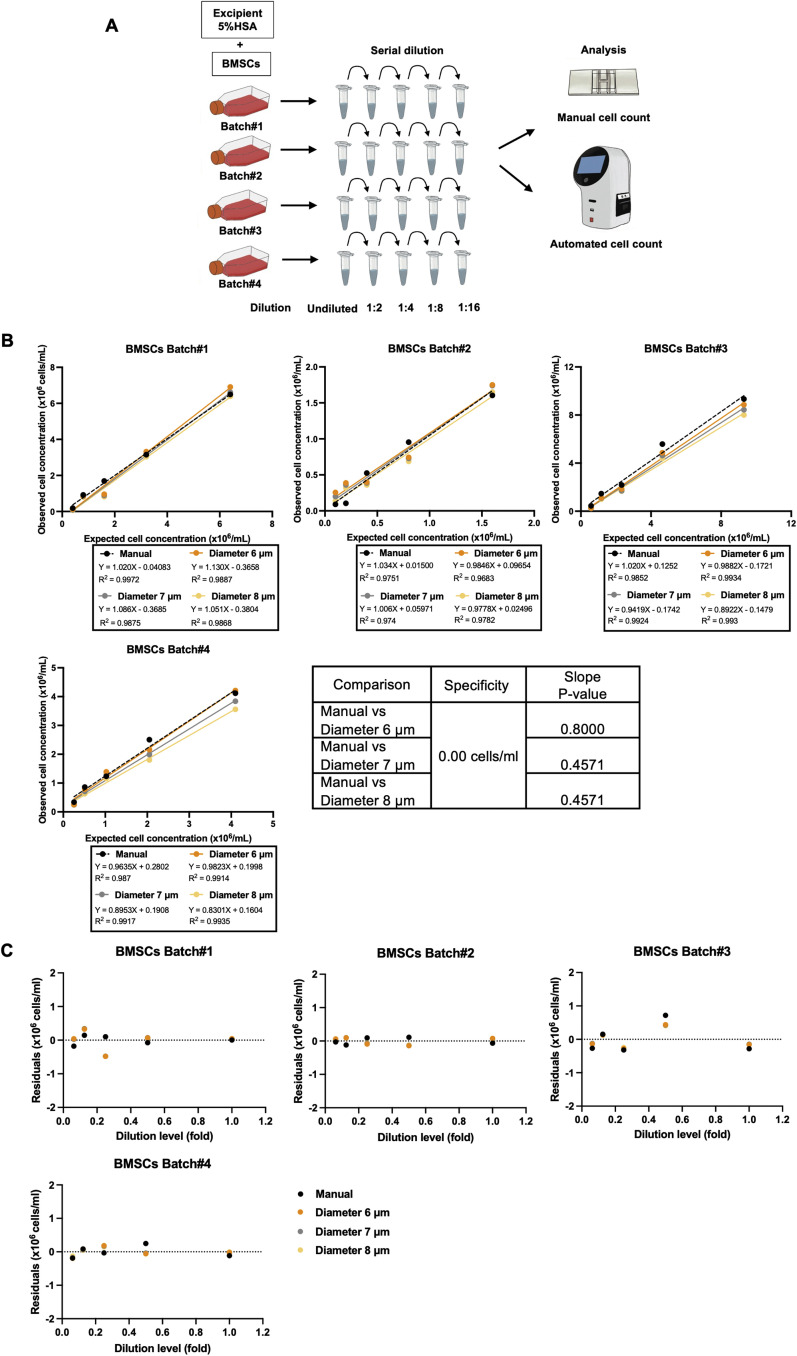
Validating diameter gating of cells counted automatically vs manually in four different BMSC batches. For automated BMSC counting, results were obtained from cell diameter gating at 6–8 µm. (A) Schematics summarizing the experimental design for total cell concentration measurement in four different BMSC batches. (B) Linear regression showing the mean value of observed total cell concentration over the value of expected total cell concentration for each BMSC batch. Table indicating specificity value and statistically significant differences of slope values between manual and automated method. (C) Residual analysis showing the difference between observed and expected total cell concentration for each batch versus fits plots. Total cell count was performed in one count by one analyst. BMSCs, bone marrow stromal cells.

The validation study was designed into four experiments (Supplementary Table 1). Experiment 1: to standardize the automated cell count, the suitable diameter and circularity parameters were evaluated by the total cell concentrations. The test was performed on serial dilutions from four different BMSC batches, using five-point dilutions (undiluted, 1:2, 1:4, 1:8, and 1:16) (Fig. 1A). To achieve the targeted dilution levels, HBSS was added to the BMSC suspension, which was then counted with an automated cell counter and a hemocytometer. Experiment 2: to analyze the best range of cell concentrations, we observed the total cell concentrations ranging between 0.1 and 8 × 10^6^ cells/mL. A stock cell solution with an estimated cell concentration of 8 × 10^6^ cells/mL was prepared. The BMSC suspension was then split into triplicates and diluted independently with HBSS in serial dilutions to reach the cell concentrations of 8, 6, 4, 2, 0.2, and 0.1 × 10^6^ cells/mL, respectively (Fig. 3A). Counts were performed by one analyst. Experiment 3: to optimize the live sensitivity parameter, the viability range (%; 2.5–100%) was evaluated. The non-viable BMSCs were added to the viable BMSC suspension in serial dilutions to achieve targeted viabilities of 100, 75, 50, 25, and 2.5% (Fig. 4A). The BMSC suspension was split into three and diluted independently, then counted by a single analyst using both methods. Experiment 4: to determine inter-operator variability, the total cell concentrations were obtained from the same BMSC suspension preparation on serial dilution starting from 4 × 10^6^ cells/mL, each performed by three different analysts (Fig. 5A). The BMSC suspension was prepared and analyzed in triplicate for each analyst.

### 2.6 Specificity

Specificity was assessed only for the automated method using the BMSC suspension matrix. The specificity test was determined on the mixture of HBSS and 0.4% trypan blue. Cell concentrations of 0.00 cells/mL and 0.0% viability were considered acceptable.

### 2.7 Accuracy

Accuracy of the automated method was evaluated by the total cell concentration and % viability using the manual method as reference values. The acceptance criterion for accuracy was within 90–110% [17]. The accuracy was performed using the following formula.

(1) $$Accuracy\;\left(\%\right)=\frac{100\;-\;\left(Tested\;value\;-\;Reference\;value\right)}{Reference\;value}x\;100$$

### 2.8 Linearity

For linearity, the expected and observed values, either the total cell concentration or % viability, were plotted. Coefficient of determination (R^2^) and slope values were reported. The acceptance criterion of R^2^ must be greater than 0.9 and the slope value must be close to 1.

### 2.9 Precision

Repeatability was the values obtained from three independent sets of the same BMSC preparation by the same analyst. Regarding intermediate precision, the values were obtained from three independent sets of the same BMSC preparation by three analysts. Both repeatability and intermediate precision were evaluated as the relative standard deviation (RSD). Setting limits for precision will depend primarily on the purpose to which the method being validated is to be put. The acceptance criterion of RSD must be <10% and the high RSD values were reported to be below 5% [18]. The precision was performed using the following formula.

(2) $$RSD\;\left(\%\right)=\frac{Standard\;deviation}{Mean}x\;100$$

### 2.10 Statistical analysis

All data were expressed as mean ± standard deviation (SD). All graphs and statistical analysis were performed by GraphPad Prism version 10.0 (GraphPad Software Inc., San Diego, USA). The Shapiro-Wilks test was used to assess the normality of the data distribution. Non-normally distributed data were analyzed using the Mann-Whitney U test for comparing two experimental groups and Kruskal–Wallis test for comparing three experimental groups. Regression was performed to assess for linearity and the range. P values <0.05 were considered as statistically significant.

## 3 Results

### 3.1 Accuracy and linearity of total cell concentration

To determine optimal parameters for automated cell counting, we first validated the effects of cell diameter gating (value: 3–10 µm) and circularity parameters (value: 50%, 75%, and 100%) on total cell concentration values. Our study has shown validation results for varying different machine parameters including cell diameter gating ranging 3–10 µm and cell circularity ranging 50–100% across four different batches. The accuracy of automated cell counting was evaluated against manual cell counts as a reference. Among four BMSC batches, the results showed high accuracy in cell diameter gating at 6 µm with 75% circularity (102.8 ± 6.0) (Supplementary Table 2). Additionally, the acceptable level of accuracy was exhibited in 6–8 µm diameter gating at 50 and 75% circularity. This result was within the average accuracy range of 90–110%, meeting the defined acceptance criterion.

**Fig. 2:**
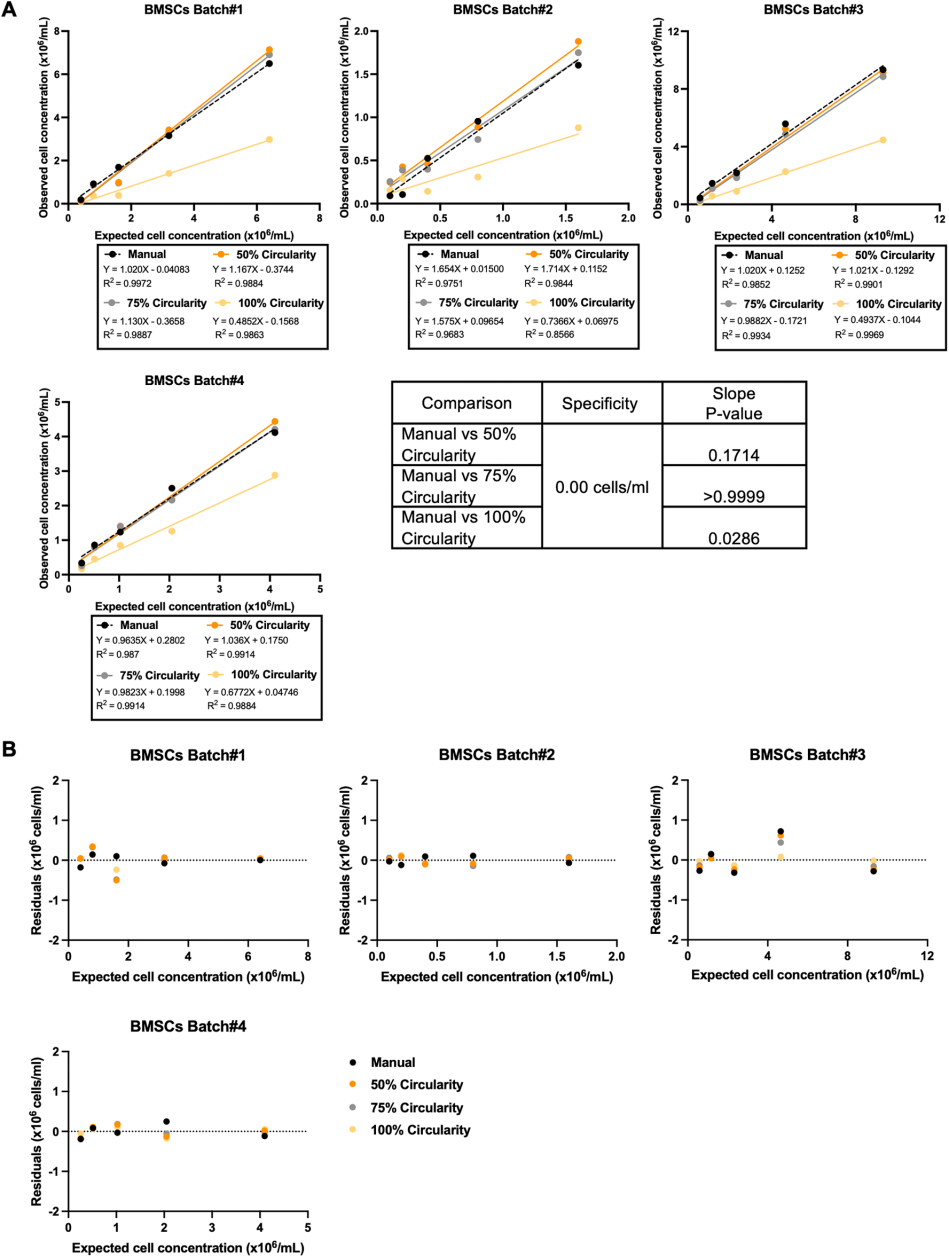
Validating circularity of cells counted automatically vs manually in four different BMSC batches. For automated BMSC counting, results were obtained at circularity levels of 50%, 75%, and 100%. (A) Linear regression showing the mean value of observed total cell concentration over the value of expected total cell concentration for each BMSC batch. Table indicating the specificity value and statistically significant differences of slope values between the manual and automated methods. (B) Residual analysis showing the difference between observed and expected total cell concentration for each batch versus fits plots. Total cell count was performed in one count by one analyst. BMSCs, bone marrow stromal cells.

Linearity validation was performed separately for each BMSC batch on cells at different concentrations within the range of 1.61–9.33 × 10^6^ cells/mL that were prepared with serial dilution (Fig. 1, Fig. 2). The results showed that the slope R^2^ was greater than 0.95 at cell diameter gating between 6 and 8 µm, and at 50% and 75% circularity. The slope obtained from automated counting at cell diameter gating of 6–8 µm was comparable to that obtained from manual count (P slope > 0.05) (Fig. 1B). Similar results were observed in automated count at 50% and 75% circularity compared to manual count (P slope > 0.05) (Fig. 2A), indicating that both methods have a comparably significant linear relationship. The specificity validation with diluent returned a cell count of 0 cells/mL, indicating that this method was specific for detecting cells without interference from the diluent (HBSS). Considering the residual values, the low variability was observed around the expected cell concentration range (Figs. 1C and 2B). Taken together, our results suggest that cell diameter gating between 6 and 8 µm and circularity at 50% and 75% provided reliable and consistent results for evaluating total cell concentrations across different BMSC batches.

### 3.2 Range of total cell concentration

**Fig. 3:**
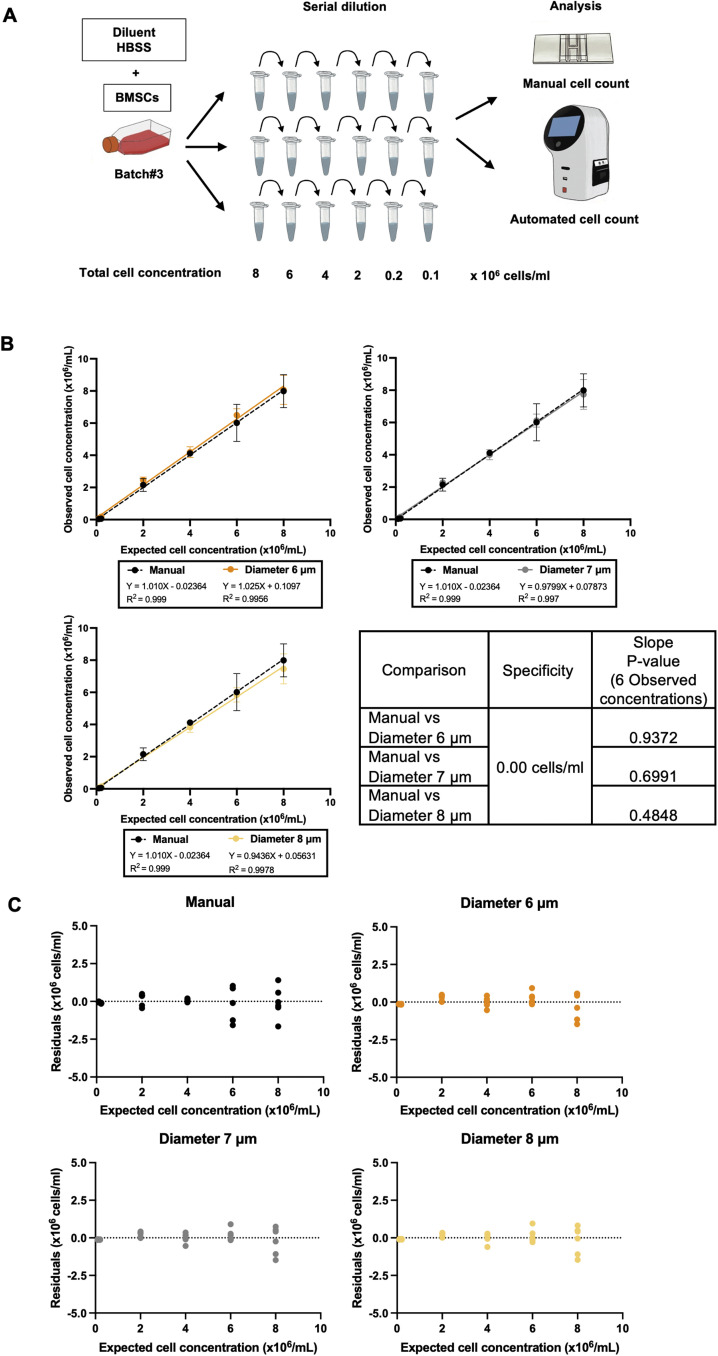
Evaluating the suitable range of the total cell concentrations over six-point concentration. manual and automated cell counting methods. (A) Schematics of the experimental design for total cell concentration measurement in a range of 0.1–8 × 10^6^ cells/ mL BMSCs. (B) Linear regression showing the mean value of observed total cell concentration over the value of expected total cell concentration for BMSCs. Table indicating the specificity value and statistically significant differences of slope values between the manual and automated methods. (C) Residual analysis showing the difference between observed and expected total cell concentration for BMSCs versus fits plots. Total cell count was performed by one analyst and calculated the mean and SD of its six counts. BMSCs, bone marrow stromal cells. SD, standard deviation.

The linearity of the observed total cell concentration against the expected total cell concentration was evaluated over six-point concentrations (0.1, 0.2, 2, 4, 6, and 8 × 10^6^ cells/mL) (Fig. 3A and Supplementary Figure 1). All R^2^ values were higher than 0.98 for the selected diameter gating (6–8 µm) and circularity parameter (75%). The slope value exhibited no significant differences between the two counting methods (P slope > 0.05) and the specificity was 0 cells/ml (Fig. 3B). For the residual analysis, both counting methods showed a low scatter of the residuals in all cell concentrations (Fig. 3C). We further examined whether or not using the selected diameter gating of 6–8 µm at circularity of 75% on the total cell concentrations in a range of 0.1–8 × 10^6^ cells/mL resulted in the average accuracy between 90 and 110% and a RSD of <10%. The automated cell counting method showed a suitable degree of accuracy and %RSD in the total cell concentrations ranging between 2 and 6 × 10^6^ cells/mL (Table 1). It is worthy of note that either increasing the cell concentration up to 8 × 10^6^ cells/mL or decreasing the cell concentration down to 1–2 × 10^5^ cells/mL increased the error.

**Table 1:** Accuracy and precision of total cell concentration at minimum cell diameter of 6–8 µm and circularity of 75%.

%Accuracy		Expected total cell concentration x 10^6^ (cells/ml)					
		8.000	6.000	4.000	2.000	0.200	0.100
Manual		100.0 ± 0.0	100.0 ± 0.0	100.0 ± 0.0	100.0 ± 0.0	100.0 ± 0.0	100.0 ± 0.0
Automated	Diameter 6 µm	**99.7 ± 11.8**	**107.3 ± 5.5**	**101.5 ± 8.1**	117.9 ± 7.1	82.7 ± 14.2	64.6 ± 24.6
	Diameter 7 µm	**95.3 ± 13.1**	**101.6 ± 6.0**	**96.7 ± 8.5**	112.0 ± 7.0	71.5 ± 10.7	45.7 ± 31.9
	Diameter 8 µm	**91.3 ± 14.5**	**96.9 ± 7.4**	**91.9 ± 9.8**	**107.0 ± 5.8**	59.6 ± 14.0	47.3 ± 34.7
Acceptable range		90.0⎯110.0%					

%RSD		Expected total cell concentration x 10^6^ (cells/ml)					
		8.000	6.000	4.000	2.000	0.200	0.100

Manual		13.9	22.3	**2.2**	20.0	73.5	71.6
Automated	Diameter 6 µm	11.8	**5.1**	**8.0**	**6.0**	17.2	38.1
	Diameter 7 µm	13.8	**6.0**	**7.8**	**6.2**	15.0	69.9
	Diameter 8 µm	15.8	**7.6**	**8.4**	**5.4**	23.6	73.3
Acceptable range		< 10%					

### 3.3 Range of viability

**Fig. 4:**
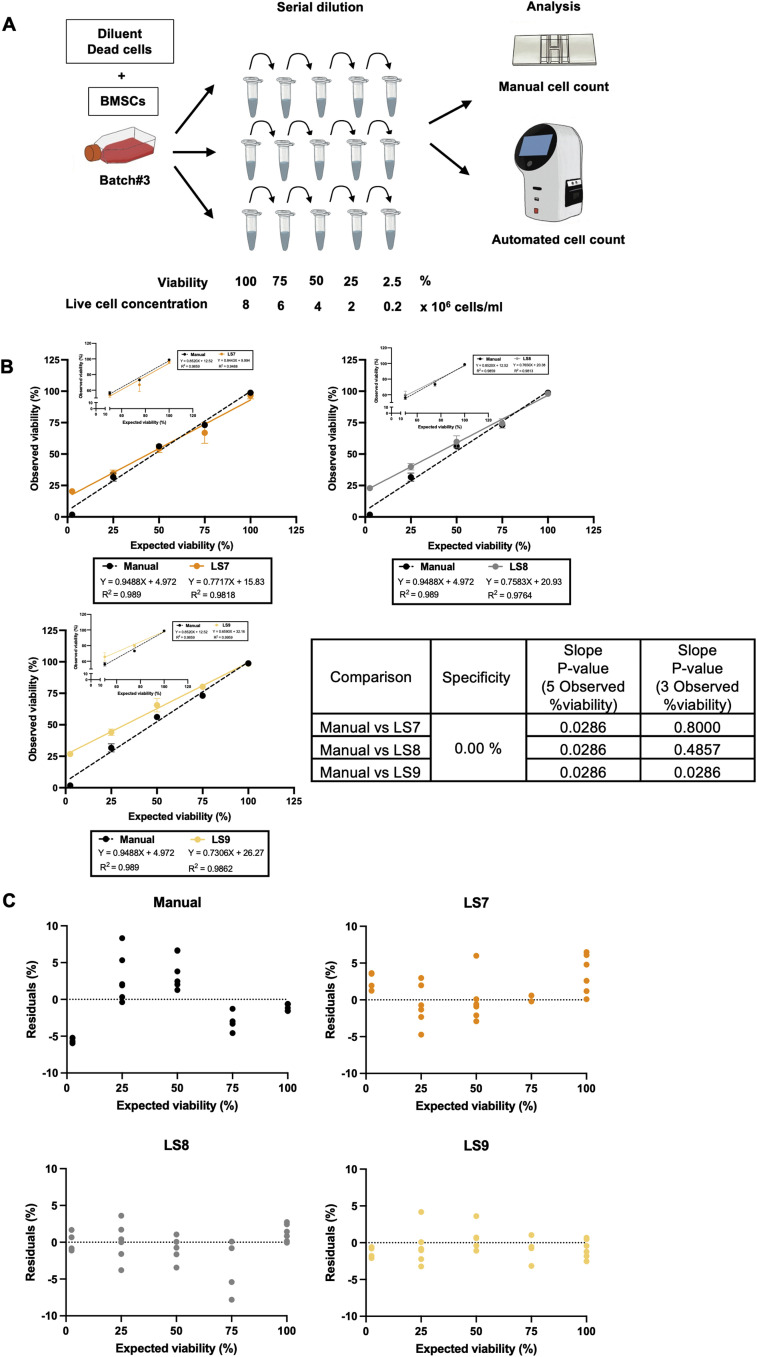
Validating live sensitivity of cells counted automatically vs manually and evaluating the suitable range of the cell viability over a five-point percentage. (A) Schematics summarizing the experimental design for viability measurement in a range of 2.5–100%. (B) Linear regression showing the mean value of observed viability over the value of expected viability for BMSCs. Table indicating the specificity value and statistically significant differences of slope values between manual and automated methods. (C) Residual analysis showing the difference between observed and expected viability for BMSCs versus fits plots. Viable cell count was performed by one analyst and calculated the mean and SD of its six counts. BMSCs, bone marrow stromal cells. SD, standard deviation.

To apply appropriate parameters for automated cell counting, we validated adjustments to the live sensitivity parameter based on cell viability. The accuracy values for live sensitivity settings at 7–9 ranged from 90 to 110%, which are considered acceptable (Supplementary Table 3). The linearity of the observed viability relative to the expected viability was further evaluated across a range of cell viabilities (2.5, 25, 50, 75, and 100%) (Fig. 4 and Supplementary Figure 2). R^2^ values were higher than 0.97, and the slope ranged from 0.7306 to 0.7717 for the selected live sensitivity (7–9) (Fig. 4B). However, the slope difference between the two counting methods was significant (P ≤ 0.05), indicating that both methods are incomparable. The specificity was 0% viability. For the residual analysis, high variability in cell viability was observed across both counting methods (Fig. 4C). The accuracy values were within 90–110%, and RSD values were below 10% in the viability range between 50 and 100% for live sensitivity at 8 (Table 2). It is remarkable that reducing the viability percentage to nearly 0% increased the error. Taken together, these findings suggest that live sensitivity at 8, with a viability range of 50–100%, consistently provides reliable viability results.

**Table 2:** Accuracy and precision of %viability at live sensitivity (LS) of 7–9.

%Accuracy		Expected viability (%)				
		100	75	50	25	2.5
Manual		100.0 ± 0.0	100.0 ± 0.0	100.0 ± 0.0	100.0 ± 0.0	100.0 ± 0.0
Automated	LS7	**97.6 ± 2.9**	**92.5 ± 13.7**	**96.2 ± 7.8**	**107.9 ± 9.9**	191.1 ± 1.8
	LS8	**99.2 ± 1.4**	**103.7 ± 6.4**	**105.4 ± 8.8**	120.4 ± 11.5	192.1 ± 1.2
	LS9	**99.7 ± 1.6**	111.9 ± 5.3	113.8 ± 7.8	128.1 ± 7.4	193.3 ± 1.2
Acceptable range		90.0⎯110.0%				

%RSD		Expected viability (%)				
		100	75	50	25	2.5

Manual		0.5	16.9	4.2	10.4	19.7
Automated	LS7	**3.0**	14.8	**8.2**	**9.2**	**1.0**
	LS8	**1.4**	**6.1**	**8.4**	**9.5**	**0.6**
	LS9	**1.6**	**4.7**	**6.8**	**5.8**	**0.6**
Acceptable range		< 10%				

### 3.4 Intermediate precision

**Fig. 5:**
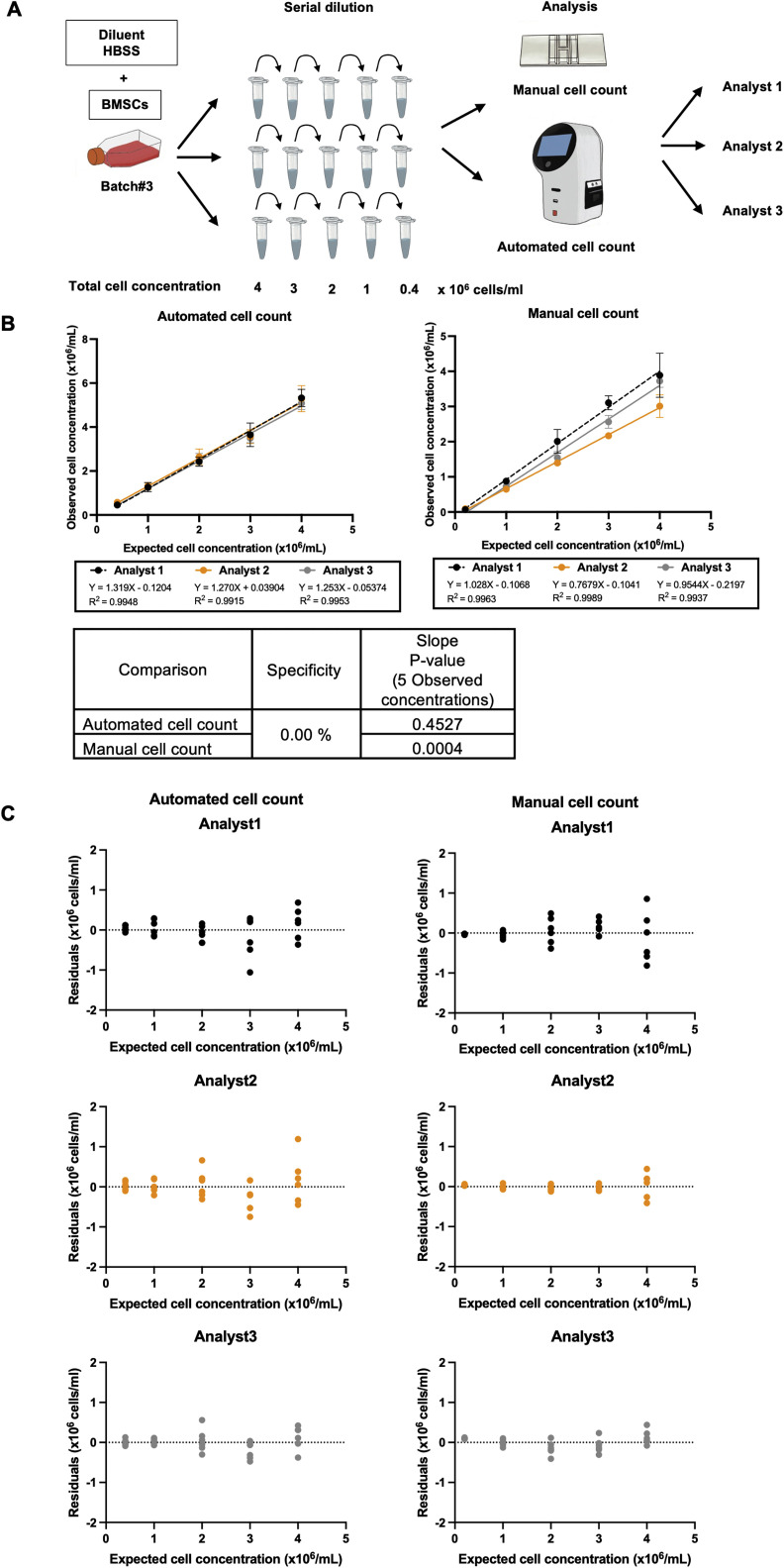
Evaluating inter-analyst variability of cells counted automatically vs manually. (A) Schematics summarizing the experimental design for total cell concentration measurement in three different analysts. (B) Linear regression showing the mean value of observed total cell concentration over the value of expected total cell concentration for each analyst. Tables indicating specificity value and statistically significant differences of slope values between manual and automated methods. (C) Residual analysis showing the difference between observed and expected total cell concentration for each analyst versus fits plots. Total cell count was performed by three different analysts and calculated the mean and SD of its six counts. BMSCs, bone marrow stromal cells. SD, standard deviation.

To compare analyst-associated variability of the automated cell count with the manual cell count, the linearity and intermediate precision of total cell concentration were determined over the range of 0.4–4 × 10^6^ cells/mL, which has been demonstrated to have accuracy and precision within the acceptable range. The R^2^ values were greater than 0.99 in all analysts for both methods. However, the slope showed a statistically significant difference among three different analysts for the manual method, with P value = 0.0004, but not for the automated method (Fig. 5B). For the residual analysis, low variability was observed regarding each analyst for both counting methods (Fig. 5C). Among three different analysts, the automated cell count showed %RSD value lower than the value obtained for the manual cell count (Table 3). These data demonstrate that the automated method yields more consistent results across different analysts.

**Table 3:** Intermediate precision of total cell concentration.

%RSD	Expected total cell concentration x 10^6^ (cells/mL)				
	4.000	3.000	2.000	1.000	0.400
Automated cell count	2.5	2.2	4.4	3.7	14.3
Manual cell count	13.2	18.0	19.4	15.5	15.7
Acceptable range	< 10%				

## 4 Discussions

The production of BMSCs for clinical use requires a quality assurance system in compliance with GMP standards for ensuring product consistency [6]. Since BMSCs are a heterogeneous cell population with unique morphology, the lack of standardized manufacturing processes may also significantly lead to batch-to-batch variation in cell products [19]. Consistent and reliable cell-counting methods are paramount for in-process quality control and batch release of ATMP products. The most widely used methods include manual cell counting using a hemocytometer and automated cell counters. However, it is important to note that the manual method may lead to less efficiency for large-scale analyses and high analyst-associated variability [20]. There has been previous studies to validate automated counting methods to enable high-throughput analysis while maintaining accuracy [8,21,22]. The validation study is designed to evaluate the specificity, linearity, range, accuracy, and precision of the test method to optimize machine parameters, including cell diameter, circularity, and live sensitivity settings.

To ensure optimal performance of the LUNA-II™ automated cell counter, it is critical to monitor total cell concentration across different BMSC batches to account for biological variability. This difference might be attributable to differences in the size and morphology of BMSCs. Herein, we established a BMSC counting protocol (diameter gating 6–8 µm, circularity 75%, and live sensitivity 8) for evaluating total cell concentration and viability. According to the predefined acceptance criteria, the accuracy of the total cell concentration and %viability was within 90–110%. Both repeatability and intermediate precision of the automated method were also confirmed to be <10%. The cell concentration and viability on human stem cells have also been measured using Countess® system with the following settings: minimum and maximum size at 8 and 60 µm, circularity at 80%, and sensitivity at 8.

The seeding density of BMSCs cultured varying between 2000 to 5000 cells/cm^2^ and filled into syringes as final product with 8 × 10^6^ cells/mL, indicating the lower and upper bounds of measurement in GMP-compliant MSC manufacturing processes. However, automated cell counting may lead to errors in the results when BMSCs are obtained at very high or very low cell concentrations. We observed the major variability at either higher (8 × 10^6^ cells/mL) or lower cell concentrations (1 × 10^5^ cells/mL). This observation raises the possibility that increased concentration may lead to cell aggregation [7]. The automated cell counter was unable to differentiate aggregated cells, thereby contributing to the decreased accuracy of cell count [21,23,24]. While the LUNA-II ™automated cell counter has an installed advanced declustering algorithm, it may not completely separate high-density, multi-layered cell clusters. Hence, we may observe clustered cells as a large single cell, leading to counting errors at higher concentrations. Over dilution, on the other hand, also undercounts cells due to erroneous analysis. Therefore, the accurate and precise range of cell concentration recommended by our study is between 2 and 6 × 10^6^ cells/mL, and BMSC concentrations outside this range will require sample adjustment to maintain counting accuracy and precision.

As cell viability is a crucial quality attribute for ensuring BMSC potency, it is important to maintain a high level of viability throughout the manufacturing process [25]. Viability has been determined from the cell suspension using the trypan blue exclusion technique. According to Food and Drug Administration (FDA) guidance, viable cells are above the accepted lower limit of 70% [26]. Our findings showed that the automated cell count achieved increased accuracy and precision at a viability range of 50–100%, covering the specified range of viability during the BMSC manufacturing process. This is in line with a previous study, which showed that high accuracy and precision were observed for viability ranging from 50 to 100% for two genetically modified prostate adenocarcinoma cell lines, CG1940 and CG8711, using the Cedex® automated cell counter [17]. There was a comparative analysis of cell viability in automated methods indicating that Vi-CELL® XR cell viability analyzer provided high accurate and precise results regarding the varying viability in CHO-K1 and U937 cells compared with Countess® system [21]. According to previous study on cultured CAR/TCR T-cell products, Vi-CELL® BLU cell viability analyzer provided accurately measuring viability, ranging 0% to 100% [13]. Only one report has studied the cell viability on human stem cells using NucleoCounter NC-100 system indicating that higher viable cell counts (> 54% viability) were correlated with less error [27]. Notably, consistent with previous reports, the variability in results obtained by the automated method increased when assessing viability near 0%, indicating lower sensitivity in detecting non-viable cells [13]. Additionally, the impact of diluents on cell viability may in part be considered. The adverse effect of phosphate-buffered saline as a diluent on cell viability was reported [28]. Consequently, HBSS was preferred as a diluent in this study owing to its superior ability to maintain cell viability.

Some limitations of our study include the lack of an appropriate reference method and the use of the trypan blue exclusion method to assess cell viability. Although the hemocytometer cell-counting method was used to assess accuracy in this study, operator-dependent variability remains a concern. Regarding cell viability assessment, trypan blue tends to overestimate the number of viable cells, as some non-viable cells rupture and disappear after staining, leading to inaccurate quantification of cell viability [29]. Moreover, the use of trypan blue dye for rapid measurement of cell viability has its limitations, since this method detects viability indirectly from membrane integrity. Trypan blue staining may not detect cells at the early stages of apoptosis, when cell injury may still be undetected. At the same time, trypan blue may struggle to distinguish small debris from dead cells, leading to overestimation of nonviable cell counts. Hence, a secondary method using the nucleic acid-binding dye PI can be performed in parallel and detected by flow cytometry, which may be beneficial to confirm the percentage of nonviable cells [30].

## 5 Conclusion

Herein, we established a BMSC counting protocol (diameter gating 6–8 µm, circularity 75%, and live sensitivity 8) for evaluating total cell concentration and viability with the LUNA ™ automated cell counter. We validated the suitability of the automated cell-counting method for quality control in BMSC manufacturing under GMP standards. The automated cell-count method showed accuracy, precision, and linearity comparable to those of the manual method. However, the suitability of certain machine parameters depends on cell type, and these parameters should be validated for each cell type.

## CRediT authorship contribution statement

**Scarlett Desclaux:** Writing – review & editing, Writing – original draft, Visualization, Validation, Methodology, Investigation, Formal analysis, Data curation, Conceptualization. **Narong Chitchongyingcharoen:** Methodology, Investigation, Formal analysis, Conceptualization. **Chanatat Threeratanah:** Investigation, Formal analysis, Conceptualization. **Chavalwit Soonthronsuk:** Methodology, Investigation, Formal analysis. **Sorawut Thamyongkit:** Investigation. **Chagriya Kitiyakara:** Supervision, Resources, Funding acquisition. **Natnaree Siriwon:** Writing – review & editing, Project administration, Methodology, Investigation, Funding acquisition, Formal analysis, Data curation, Conceptualization.

## Declaration of competing interest

The authors declare that they have no known competing financial interests or personal relationships that could have appeared to influence the work reported in this paper.

## Data Availability

Data will be made available on request.

## Appendix Supplementary materials

Image, application 1
