# Supplementary material for: Validation of an automated total and viable cell counting for GMP-compliant production of human bone marrow stromal cells
Source: Biotechnol Rep (Amst). 2026 Jul 27;51:e00974. doi: 10.1016/j.btre.2026.e00974 (PMC13453019; doi:10.1016/j.btre.2026.e00974)
Supplement: Supplementary file 1 [file mmc1.docx]

**Supplementary Table 1: Workflow summarizing the validation studies of automated cell counter.**

| Experiment design | Parameter for validation | Definition | Validation assay |
| --- | --- | --- | --- |
| Experiment 1 | Cell diameter and circularity | Five-point dilutions:  undiluted, 1:2, 1:4, 1:8, and 1:16 in four different batches (n=1) | Specificity, accuracy, linearity, and precision (repeatability) |
| Experiment 2 | Cell diameter | Six-point concentrations:  8, 6, 4, 2, 0.2, and 0.1 x 10^6^ cells/mL (n=6) | Specificity, accuracy, linearity, and precision (repeatability) |
| Experiment 3 | Live sensitivity | Five-point viability percentages:  100, 75, 50, 25, and 2.5% (n=6) | Specificity, accuracy, linearity, and precision (repeatability) |
| Experiment 4 | Analyst-associated variability | Five-point concentrations:  4, 3, 2, 1, and 0.4 x 10^6^ cells/mL (n=6) | Specificity, linearity, intermediate precision |

**Supplementary Table 2: Accuracy of total cell concentration at minimum cell diameter of 3–10 µm and circularity of 50–100%**

| %Accuracy | | BMSC batch | | | | |
| --- | --- | --- | --- | --- | --- | --- |
|  |  | #1 | #2 | #3 | #4 | Average |
| Manual | | 100.0 | 100.0 | 100.0 | 100.0 | 100.0 ± 0.0 |
| Diameter 3 µm | 50% Circularity | 115.1 | 118.9 | **107.6** | 126.5 | 117.0 ± 7.9 |
| Diameter 4 µm |  | 113.6 | 118.1 | **105.2** | 122.2 | 114.8 ± 7.3 |
| Diameter 5 µm |  | 111.3 | 116.4 | **102.4** | 115.0 | 111.3 ± 6.3 |
| Diameter 6 µm |  | **109.0** | 114.6 | **97.7** | **107.3** | **107.2 ± 7.0** |
| Diameter 7 µm |  | **105.7** | 113.2 | **93.5** | **97.6** | **102.5 ± 8.8** |
| Diameter 8 µm |  | **102.0** | **109.8** | 86.9 | 89.7 | **97.1 ± 10.7** |
| Diameter 9 µm |  | **97.6** | **108.8** | 82.9 | 79.7 | **92.2 ± 13.5** |
| Diameter 10 µm |  | **93.4** | **105.0** | 78.0 | 68.5 | 86.2 ± 16.2 |
| Acceptable range (%) | | 90.0⎯110.0 | 90.0⎯110.0 | 90.0⎯110.0 | 90.0⎯110.0 | 90.0⎯110.0 |

| %Accuracy | | BMSC batch | | | | |
| --- | --- | --- | --- | --- | --- | --- |
|  |  | #1 | #2 | #3 | #4 | Average |
| Manual | | 100.0 | 100.0 | 100.0 | 100.0 | 100.0 ± 0.0 |
| Diameter 3 µm | 75% Circularity | 111.5 | 112.8 | **105.3** | 124.5 | 113.5 ± 8.0 |
| Diameter 4 µm |  | **109.4** | 111.8 | **101.7** | 118.0 | 110.2 ± 6.8 |
| Diameter 5 µm |  | **107.9** | 110.8 | **99.1** | 112.4 | **107.6 ± 5.9** |
| Diameter 6 µm |  | **106.0** | **108.3** | **94.7** | **102.3** | **102.8 ± 6.0** |
| Diameter 7 µm |  | **101.9** | **107.8** | 89.5 | **92.8** | **98.0 ± 8.4** |
| Diameter 8 µm |  | **98.4** | **102.7** | 83.4 | 84.4 | **92.2 ± 9.8** |
| Diameter 9 µm |  | **95.1** | **100.9** | 77.9 | 75.7 | 87.4 ± 12.5 |
| Diameter 10 µm |  | 89.9 | **97.1** | 72.5 | 64.2 | 80.9 ± 15.2 |
| Acceptable range (%) | | 90.0⎯110.0 | 90.0⎯110.0 | 90.0⎯110.0 | 90.0⎯110.0 | 90.0⎯110.0 |

| %Accuracy | | BMSC batch | | | | |
| --- | --- | --- | --- | --- | --- | --- |
|  |  | #1 | #2 | #3 | #4 | Average |
| Manual | | 100.0 | 100.0 | 100.0 | 100.0 | 100.0 ± 0.0 |
| Diameter 3 µm | 100% Circularity | 10.1 | 31.4 | 30.7 | **98.9** | 42.8 ± 38.7 |
| Diameter 4 µm |  | 3.2 | 30.5 | 19.5 | 87.9 | 35.3 ± 36.8 |
| Diameter 5 µm |  | 6.9 | 25.4 | 10.4 | 73.8 | 29.1 ± 30.9 |
| Diameter 6 µm |  | 18.0 | 17.8 | 8.7 | 57.1 | 25.4 ± 21.6 |
| Diameter 7 µm |  | 31.1 | 8.5 | 29.8 | 36.1 | 26.4 ± 12.2 |
| Diameter 8 µm |  | 41.5 | 1.9 | 49.5 | 3.1 | 24.0 ± 25.0 |
| Diameter 9 µm |  | 56.7 | 7.9 | 62.1 | 23.6 | 37.6 ± 26.1 |
| Diameter 10 µm |  | 76.4 | 30.6 | 78.5 | 57.2 | 60.7 ± 22.2 |
| Acceptable range (%) | | 90.0⎯110.0 | 90.0⎯110.0 | 90.0⎯110.0 | 90.0⎯110.0 | 90.0⎯110.0 |

**Supplementary Table 3: Accuracy of %viability at live cell sensitivity of 1–9**

|  | %Accuracy |
| --- | --- |
| Manual | 100.0 ± 0.0 |
| LS1 | 487.7 ± 399.7 |
| LS2 | 806.7 ± 912.4 |
| LS3 | 452.8 ± 553.0 |
| LS4 | 114.3 ± 112.4 |
| LS5 | 62.7 ± 73.0 |
| LS6 | 30.7 ± 26.1 |
| LS7 | **92.1 ± 15.4** |
| LS8 | **103.2 ± 5.1** |
| LS9 | **106.4 ± 2.0** |
| Acceptable range (%) | 90.0⎯110.0 |

**Supplementary Figure 1:** Representative brightfield images showing cell detection from the concentration of 0.1–8 million cells/ml. Green circles tag for the viable cells and red circles tag for non-viable cells.

**Supplementary Figure 2:** Representative brightfield images showing cell detection from the viability of 2.5–100%. Green circles tag for the viable cells and red circles tag for non-viable cells.
